# High-throughput screening identifies inhibitors of DUX4-induced myoblast toxicity

**DOI:** 10.1186/2044-5040-4-4

**Published:** 2014-02-01

**Authors:** Darko Bosnakovski, Si Ho Choi, Jessica M Strasser, Erik A Toso, Michael A Walters, Michael Kyba

**Affiliations:** 1 Lillehei Heart Institute, 312 Church St. SE, Minneapolis, MN 55455, USA; 2 Department of Pediatrics, University of Minnesota, Nils Hasselmo Hall, 312 Church St. S.E, Minneapolis, MN 55455, USA; 3 Institute for Therapeutics Discovery and Development, University of Minnesota, 717 Delaware St. SE, Minneapolis, MN 55455, USA; 4 Present Address: Faculty of Medical Sciences, University Goce Delčev - Štip, Krste Misirkov b.b, 2000 Štip, R. Macedonia

**Keywords:** Facioscapulohumeral muscular dystrophy, DUX4, Small molecule inhibitors, High-throughput screening

## Abstract

**Background:**

Facioscapulohumeral muscular dystrophy (FSHD) is caused by epigenetic alterations at the D4Z4 macrosatellite repeat locus on chromosome 4, resulting in inappropriate expression of the DUX4 protein. The DUX4 protein is therefore the primary molecular target for therapeutic intervention.

**Methods:**

We have developed a high-throughput screen based on the toxicity of DUX4 when overexpressed in C2C12 myoblasts, and identified inhibitors of DUX4-induced toxicity from within a diverse set of 44,000 small, drug-like molecules. A total of 1,280 hits were then subjected to secondary screening for activity against DUX4 expressed by 3T3 fibroblasts, for absence of activity against the tet-on system used to conditionally express DUX4, and for potential effects on cellular proliferation rate.

**Results:**

This allowed us to define a panel of 52 compounds to use as probes to identify essential pathways of DUX4 activity. We tested these compounds for their ability to protect wild-type cells from other types of cell death-inducing insults. Remarkably, we found that 60% of the DUX4 toxicity inhibitors that we identified also protected cells from tert-butyl hydrogen peroxide, an oxidative stress-inducing compound. Compounds did not protect against death induced by caspase activation, DNA damage, protein misfolding, or ER stress. Encouragingly, many of these compounds are also protective against DUX4 expression in human cells.

**Conclusion:**

These data suggest that oxidative stress is a dominant pathway through which DUX4-provoked toxicity is mediated in this system, and we speculate that enhancing the oxidative stress response pathway might be clinically beneficial in FSHD.

## Background

Facioscapulohumeral muscular dystrophy (FSHD) is one of the most common degenerative myopathies. It is caused by epigenetic alterations within D4Z4, the macrosatellite repeat at the end of the long arm of chromosome 4 [1-4], which arise due to contractions in repeat number [5,6] or second site mutation of regulators of D4Z4 [7]. FSHD only arises on a specific ‘pathogenic’ allele of chromosome 4, termed 4qA161 [8,9], the key feature of which is the presence of a polyadenylation signal [10,11]. This indicates that an mRNA transcript produced from D4Z4 must be polyadenylated to cause disease. The D4Z4 transcript encodes the DUX4 protein, a double-homeodomain presumptive transcription factor [12] which kills myoblasts and other cells when expressed at high levels [13,14]. DUX4 and homologues of DUX4 also have effects when expressed in myoblasts at low, non-toxic levels: MyoD expression is lost and cells lose the ability to differentiate into myotubes [13,15,16]. Because of these pathological activities, DUX4 is the key molecular target in the development of a pharmacological treatment for FSHD.

We have previously developed a cell line in which DUX4 can be induced to various levels of expression with doxycycline (dox) [13]. Here, we use the cell death phenotype as a screening tool to identify compounds from within a diverse chemical library that interfere with DUX4. Because the phenotype requires both activity of the DUX4 protein and transmission of a signal through one or more cell death pathways, we reasoned that such a screen would identify both compounds that interfere directly with the activity of the DUX4 protein, as well as compounds that interfere with essential DUX4 downstream pathways leading to cell death. Since these pathways are not well understood, such compounds could serve as useful probes into the molecular mechanism by which DUX4 causes cell death.

## Methods

### Composition of the library

We screened a 44,000-compound subset of the UT Southwestern chemical library. Contained within this library are sub-libraries created by Chemical Diversity (San Diego, CA, USA), Chembridge (San Diego, CA, USA), and ComGenex (Budapest, Hungary). The library also contains smaller chemical collections, notably the Prestwick library of 1,100 off-patent FDA-approved compounds.

#### Cell culture

C2C12 cells and the inducible C2C12 cell lines (iC2C12-DUX4 and iC2C12-Luc) were cultured in proliferation medium comprising high glucose Dulbecco’s Modified Eagle Media (DMEM) with L-glutamine and sodium pyruvate (Gibco), penicillin and streptomycin (P/S, Gibco), and 20% fetal bovine serum (FBS, Atlanta Biologicals) at 37°C in 5% O_2_/5% CO_2_. The i3T3-DUX4 cell line was cultured in DMEM, P/S, and 10% FBS.

### Luciferase assay

iC2C12-luc cells were plated in 384 well plates at 1,000 cells per well. The following day the luciferase was induced with 500 ng/mL dox. Luminescence was detected by the Bright-Glo™ Luciferase Assay System (Promega, Maddison, WI, USA).

### High-throughput screen (HTS)

A single cell subclone of iC2C12-DUX4 cells was expanded and frozen into 80 vials for screening. A large frozen stock of the cells at the same growth stage was necessary to minimize variation in cell viability. iC2C12-DUX4 cells were plated in 384-well dishes at a density of 1,000 cells per well. One day after plating, compounds were added at a concentration of 5 μM (diluted in 1% DMSO) followed by doxycycline at 500 ng/mL. The ATPlite assay (Perkin Elmer, Waltham, MA, USA) measuring cell viability was performed 24 h later according to the manufacturer’s specifications.

### Cell death inhibition assays

C2C12 cells were seeded in 25 μL of medium at 625 cells/well in 384 well plates using a Biomek FX robot (Beckman, Indianapolis, IN, USA). The cells were allowed to attach for 24 h before adding cell death inducing reagents: Ionomycin (12.5 μM; Cayman, Ann Arbor, MI, USA), Staurosporine (0.0125 μM; Cayman), tert-butyl-hydroperoxide (tBHP; 25 μM; Sigma), ABT-263 (12.5 μM; Selleckchem, Houston, TX, USA), Etoposide (12.5 μM; Cayman), and Tunicamycin (2.5 μM; Cayman) using an ECH 550 robot (LabCyte, Sunnyvale, CA, USA). The plates were then incubated for 24 h and cell viability was measured using the CellTiter-Glo luminescent assay based on quantitation of ATP (Promega, Maddison, WI, USA). Briefly, the plates were equilibrated at room temperature and media was removed. CellTiter-Glo reagent diluted (1:1) in PBS was added and the plates were read in the Analyst AD 96/384 plate reader (LJL Biosystem, Sunnyvale, CA, USA).

### Dose response analyses

For dose response analysis against DUX4, iC2C12-DUX4 cells were treated with compounds at different doses (7.44 μM, 2.47 μM, 0.81 μM, 0.27 μM, 0.09 μM, 0.03 μM, 0.01 μM) followed by dox at 250 ng/mL. For dose response against tBHP, C2C12 cells were treated with compounds at different dose (7.44 μM, 2.47 μM, 0.81 μM, 0.27 μM, 0.09 μM, 0.03 μM, 0.01 μM) followed by tert-butyl-hydroperoxide (tBHP; 25 μM). The plates were then incubated for 24 h and cell viability was measured using CellTiter-Glo.

### DUX4 transfection in 293 T cells

A total of 293T cells were plated in 96 well plates and transfected with 200 ng of pClneo-DUX4 plasmids using TransIT-LT1 transfection reagent (Mirus). Cells were treated with compounds 6 h after transfection and cell viability measured using CellTiter-Glo luminescent assay 24 h after compound treatment.

## Results

### Development of the primary screen

**Figure 1:**
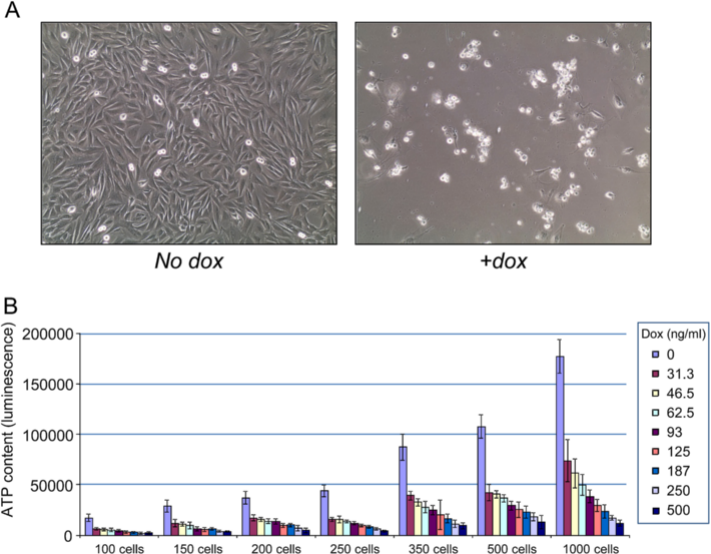
**Development of the primary assay. (A)** Cell death of iC2C12-DUX4 cells 36 h after addition of 500 ng/mL doxycycline. **(B)** Optimization of cell number and dox concentration. The clearest difference between viability of control and DUX4-induced cells was seen at 1,000 cells per well with 500 ng/mL dox. *n* = 6. Error bars represent SEM.

Dox-induced DUX4 expression provoked multiple changes, both obvious and subtle, to C2C12 myoblasts, resulting in cell death above a certain threshold, with death occurring more rapidly at higher concentrations of dox. Above 125 ng/mL dox, induction caused cell death within 24 h. Toxic effects can be observed after 6-8 h of induction, and become more prominent with time. At 36 h post induction a majority of the cells (>95%) are dead and have lifted from the plate (Figure 1A) and the few cells still attached exhibit severe morphological abnormalities and are not able to proliferate. We used an assay based on colorimetric detection of ATP levels in the cells to quantify viability after DUX4 expression, and optimized cell number, dox concentration, and time of exposure, to develop a robust high-throughput screening assay with a Z-factor value of 0.74 (*n* = 8) (Figure 1B).

### High-throughput screening (HTS): Prestwick Library

**Figure 2:**
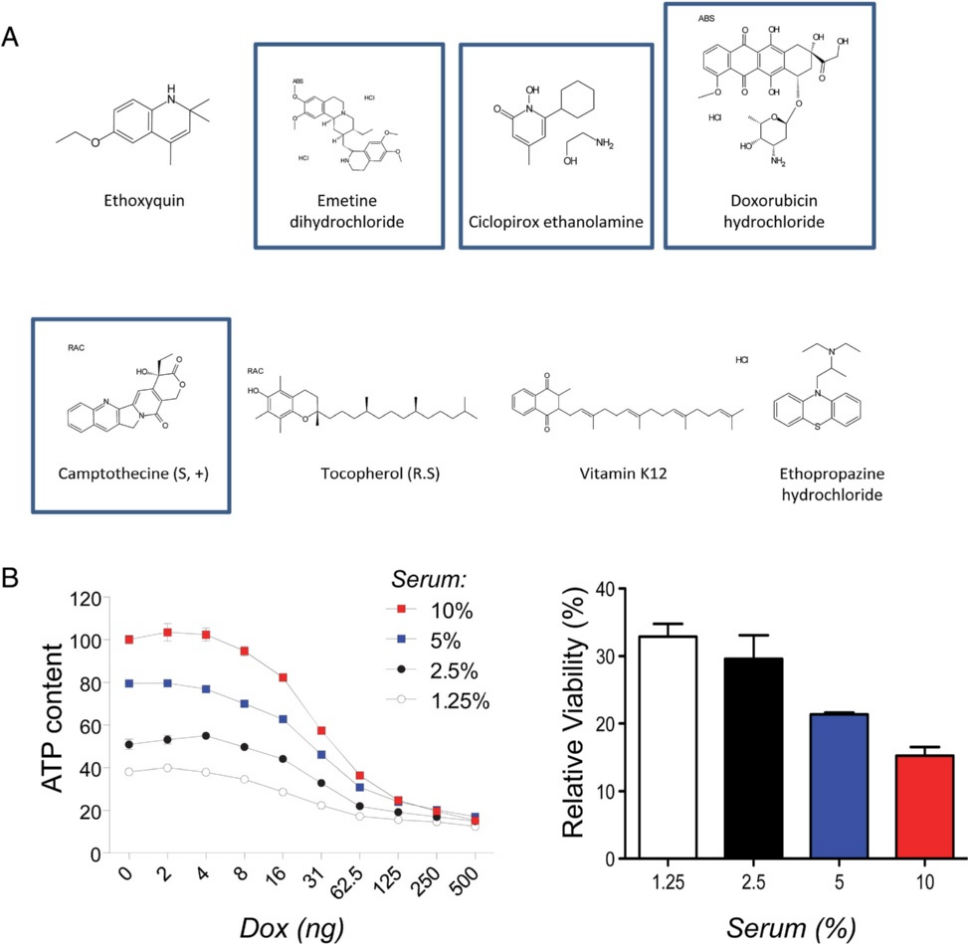
**Compounds from the Prestwick library. (A)** Prestwick compounds identified in the screen. Boxes indicate compounds with antimitotic activity. **(B)** Growth rate-dependence of toxicity. Left: 24-h toxicity of DUX4 against cells growing in media of different serum concentrations. Right: Toxicity of DUX4 expressed as relative viability at 500 ng/mL dox compared to no dox. Note that as cells grow more slowly, the relative difference in viability between control and DUX4-induced cells diminishes.

Using this system, we performed an initial test screen of 1,120 FDA-approved off-patent compounds (Prestwick Chemical, France). This screen identified eight compounds, of which several were classified as antimitotics (Figure 2A). Since the screen was based on an indirect assay for live cells, an antimitotic activity seemed counterintuitive. We therefore considered the possibility that DUX4 kills rapidly proliferating cells more effectively than slowly proliferating or non-mitotic cells. To test this, we exposed C2C12 cells to different concentrations of serum, which resulted in different rates of growth, and exposed these cells to different concentrations of dox. We found that the IC50 of dox was not changed significantly when cells were growing more slowly (Figure 2B), however the extent of cell killing was less complete at 24 h when cells were growing more slowly. This indicated that antimitotic compounds would be a class of false positives from this screen, suggesting the importance of secondary screening for effects on cell proliferation.

### HTS on a large synthetic library

**Figure 3:**
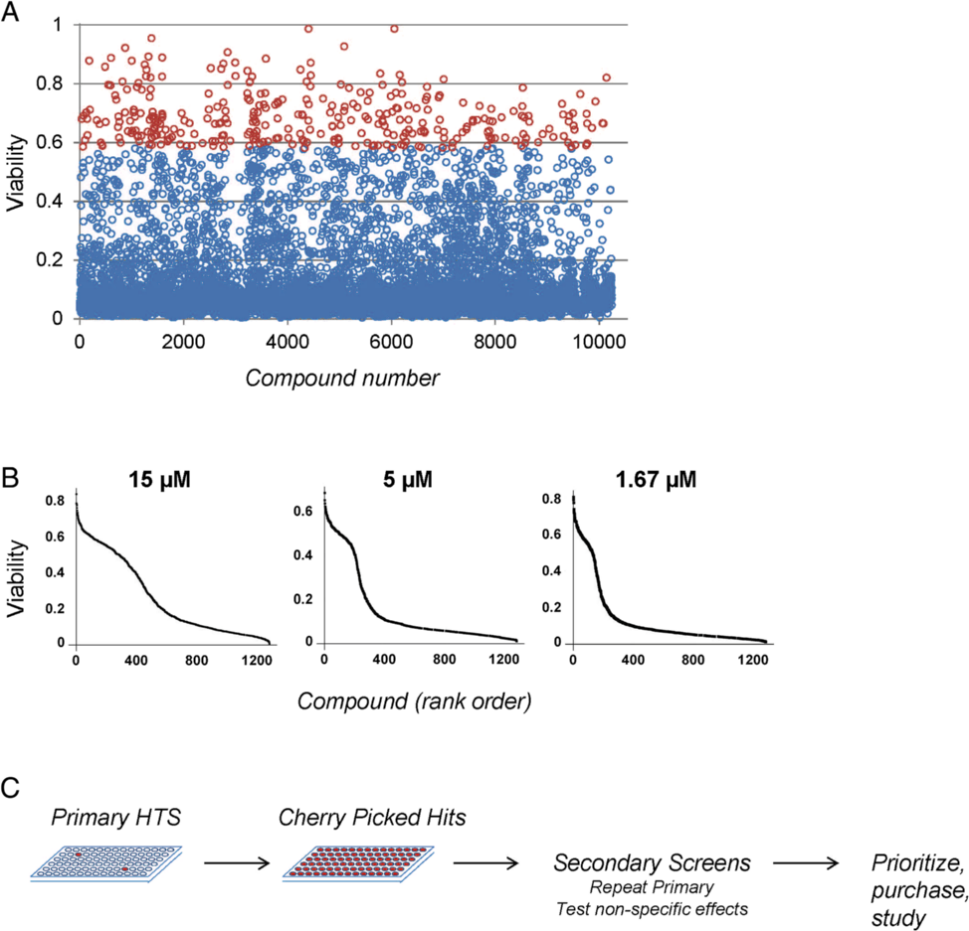
**Primary screen of 44,000 compounds and confirmation of activity. (A)** Example screening results for the first approximately 10 K compounds. Viability of cells exposed to 500 ng/mL dox and 5 μM compound is indicated on the y-axis, normalized to control wells, not treated with doxycycline. Red circles indicate viability scores ≥3 SD above the mean viability. **(B)** Retesting of compounds at 1.67 μM, 5 μM, and 15 μM. **(C)** Schematic of workflow. Primary HTS identified rare ‘hits’, which were then collected in very small volumes into cherry-picked plates. These were then used for secondary screens. Using data from the secondary screens, individual compounds are identified for deeper study.

We then applied the high throughput screen to an additional 43,000 compounds created specifically for pharmacological screening using automated design algorithms that exclude structures that are likely to be reactive, precipitate easily or be insoluble, and that comprise structures that are small and synthetically accessible (Figure 3A). We identified a total of 1,280 compounds that showed a difference in viability approximately 3 standard deviations above the mean control value when added to the culture medium at 5 μM, and cherry-picked these for secondary screening. Above 3 standard deviations, random chance predicts that approximately 1% of inactive compounds will be selected, therefore our first secondary assay was to retest each compound (at 3 different concentrations 1.67, 5, and 15 μM; Figure 3B). Slightly under half of the cherry-picked compounds showed activity when retested at least one of these concentrations.

### Secondary phenotypic screens

**Figure 4:**
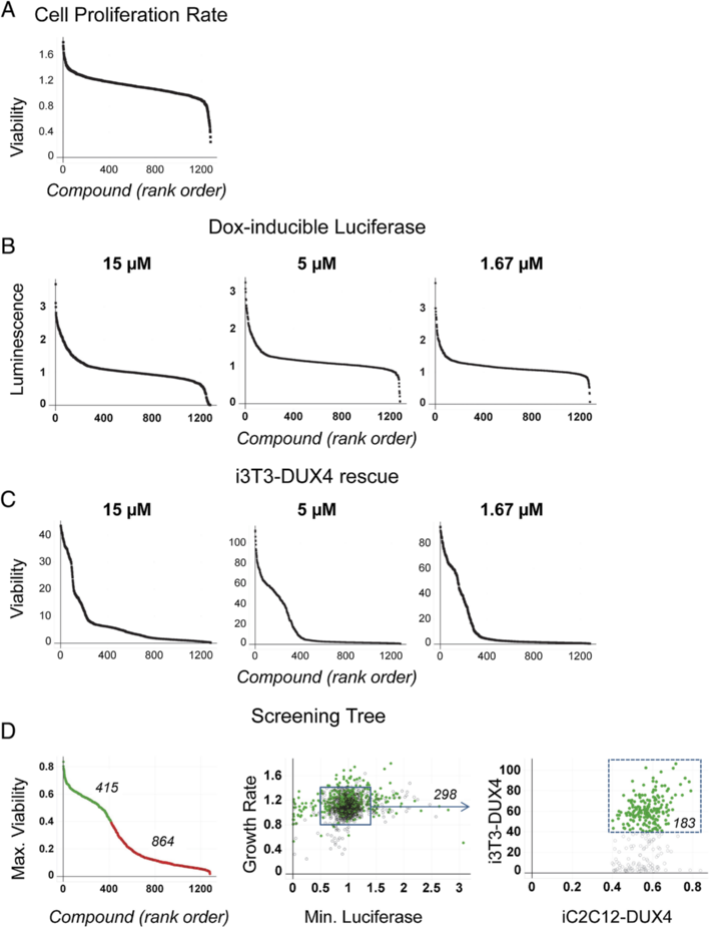
**Secondary screens. (A)** Effects of compounds on cell proliferation. **(B)** Effects of compounds on a dox-inducible luciferase transgene. Compounds inhibiting luciferase expression are predicted to also inhibit DUX4 expression and thus give a false positive increase in viability. **(C)** Protection of an independent cell line (i3T3-DUX4) from cell death induced by DUX4. **(D)** Screening tree. At left is the graphed the maximum viability at any dose of each compound in the repeat of the primary screen. Green circles indicate compounds considered to have passed this screen (that is, repeated the primary screen by protecting cells against DUX4), and the activity of these compounds is indicated in the following panel. The middle panel shows the activity of the selected compounds on the first two secondary screens: effect on growth rate shown on the y-axis, and inhibition of luciferase (minimum luciferase expression at any concentration of compound) on the x-axis. All compounds are plotted, those with activity in the previous assay are shown as green circles, those that failed the previous assay are shown as gray circles. The box indicates cutoffs set for growth rate and dox-inducible luciferase assays. The third panel shows the activity of selected compounds (that is, those within the box, which did not alter growth rate or affect dox-inducible luciferase expression levels) against DUX4-expressing 3 T3 cells on the y-axis *vs.* the repeat of the primary assay (same data as the far left panel) on the x-axis. Compounds indicated in green protect against DUX4 independent of cell type.

We then tested each compound in a number of secondary screens designed to eliminate different classes of false positives. We first tested for effects on C2C12 cell proliferation using WT unmodified C2C12 cells. Approximately 34 compounds inhibited proliferation when applied at the dose used in the primary screen (Figure 4A). We also eliminated 44 compounds that caused elevated scores on the ATP assay with WT C2C12 cells (Figure 4A).

We anticipated that certain compounds might protect cells by interfering with the dox-inducible system, and thereby prevent DUX4 from being expressed when cells were treated with dox. To identify such compounds, we tested each compound in C2C12 cells modified to express luciferase from the identical genomic locus in which DUX4 was expressed [13]. This identified 52 compounds that inhibited luciferase expression after 24 h of dox treatment, which therefore act by inhibiting the dox-inducible system (Figure 4B).

To eliminate compounds that acted on cell-type specific pathways, we then screened all compounds at three concentrations (1.67, 5, and 15 μM) on 3T3 cells modified to express DUX4 in response to dox. Initial cell number, induction time, and concentration of dox were optimized for the new cell line. Slightly over 60% of compounds identified as active in protecting C2C12 cells from DUX4 also protected 3T3 cells from DUX4 to a level of 3 standard deviations above the untreated control in at least one concentration (Figure 4C).

### Identification of chemical series and purchase of selected compounds

**Figure 5:**
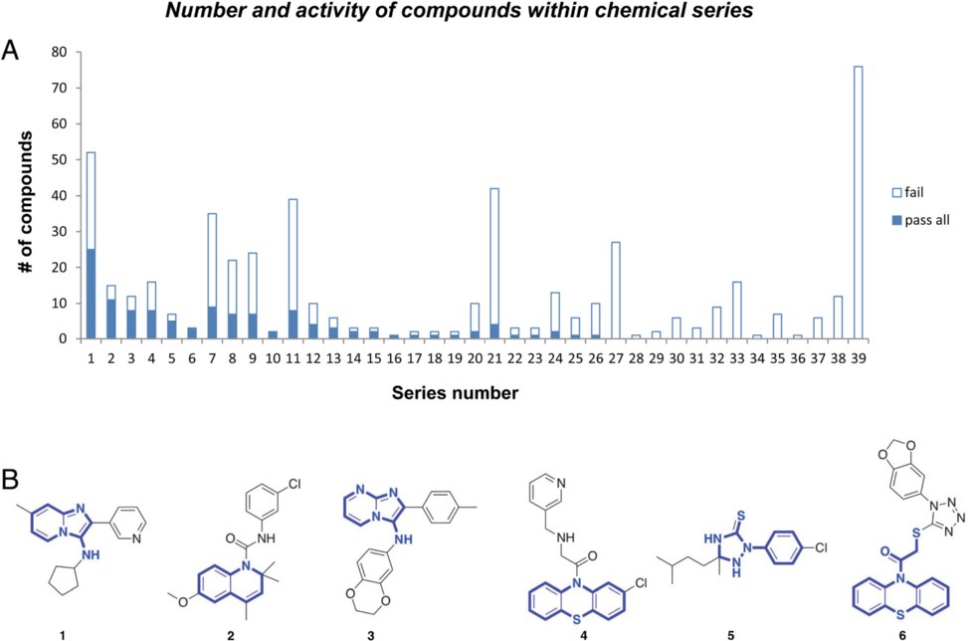
**Chemical series identified in the set of specifically active compounds. (A)** Number of compounds in each chemical series. **(B)** Structures of selected compounds from the top six chemical series. The core that defines the chemical series is shown in blue.

**Figure 6:**
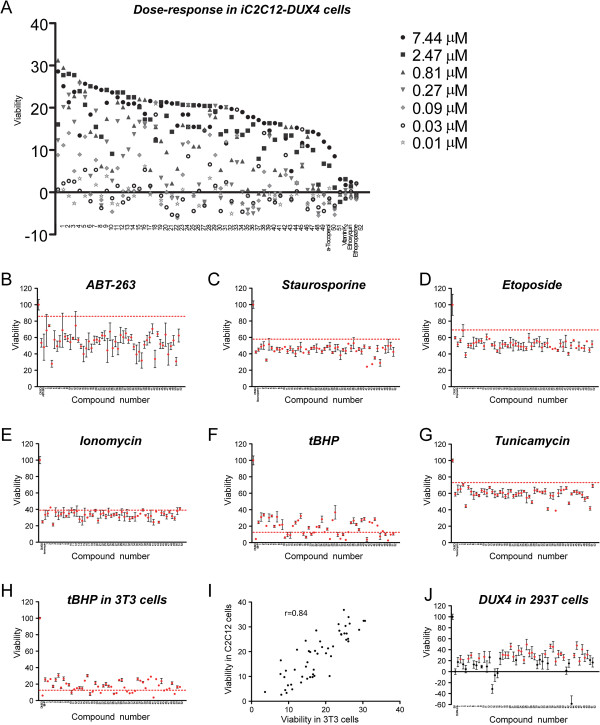
**Activity of repurchased compounds on cell death induced by DUX4 or other cytotoxic pathways. (A)** Viability of DUX4-expressing C2C12 cells exposed to various concentrations of compounds, from 0.01 μM to 7.44 μM. Compounds are arranged in order of greatest viability at any concentration. Four Prestwich compounds are also shown: α-tocopherol, vitamin K2, ethoxyquin, and ethopropazine HCl. **B-G**: Viability of C2C12 cells exposed to various cell death-inducing compounds in the presence of 5 μM compounds 1 to 52 (the active compounds of the 54 that were purchased). The Y axis represents viability (ATP content). The first point in each series represents untreated cells, the second represents cells treated with toxic agent alone, and the remaining points represent cells treated with toxic agent plus inhibitory compounds, in order from 1 to 52 (*n* = 3), error bars = SEM. The dashed red line represents 3 standard deviations above the control sample without compound. **(B)** Protection from ABT-263. **(C)** Protection from Staurosporine. **(D)** Protection from Etoposide. **(E)** Protection from Ionomycin. **(F)** Protection from tBHP. **(G)** Protection from Tunicamycin. **(H)** Viability of 3T3 cells exposed to tBHP in the presence of compounds 1 to 52. **(I)** Protection from tBHP in C2C12 cells *vs.* 3T3 cells. R = Spearman’s correlation coefficient. The strong correlation indicates that compounds tend to protect equally well in both cell types. **(J)** Activity of 52 repurchased compounds on cell death induced by DUX4 in 293 T cells. Viability of 293T cells transfected with vector control (EV), DUX4 plus carrier (DMSO) alone, or DUX4 and treated with of 5 μM compounds 1 to 52 (*n* = 6). The Y axis represents improvement in viability over DUX4 transfection alone. Red dots represent compounds that gave a statistically significant (*P* <0.05) improvement in viability over DUX4 treatment with carrier alone.

After eliminating those compounds that did not repeat in the primary assay and those that failed various secondary assays, we were left with 183 compounds. The pattern of loss to various assays is summarized in Figure 4D. From the original hit set, we identified 39 chemical series. Those compounds that passed through all of the secondary screens represented 26 chemical series, comprising 120 compounds and 63 singlet compounds with no structurally similar compound within the cherry-picked set. These chemical series are outlined in Figure 5. Interestingly, one of the eight Prestwick compounds, ethoxyquin, an antioxidant, falls into series 2. We selected 54 compounds to purchase, prioritizing those within chemical series, those with desirable medicinal chemical properties or otherwise interesting structures. We identified a number of compounds that had structures known to be associated with interference with multiple assays (pan-assay interfering compounds, PAINS [17]) and we specifically avoided these. We verified the identity of each purchased compound by mass spectrometry, and tested each of these in the primary DUX4-mediated cell death assay over a range of concentrations. With the exception of two compounds that failed identity/purity, all compounds demonstrated activity against DUX4-induced C2C12 cell death (Figure 6A, Additional file 1: Table S1). Interestingly, four of the top eight compounds (in terms of maximum viability at any dose) were from chemical series 2.

### Activity of the panel of compounds against other cell death insults

Besides compounds that interfere directly with the activity of a protein, a cell-based screen can identify compounds that interfere upstream, with pathways necessary for activity of the protein, or downstream, with pathways that are activated by the protein. We reasoned that because the primary screen was based on cell death, it might have recovered mainly antiapoptotic compounds. We therefore tested the panel of 52 selected compounds for protection against a variety of cell-death inducing stimuli, beginning with ABT-263 a compound that induces apoptosis directly by inhibiting Bcl2 [18]. No compounds were able to interfere with apoptosis induced by interfering directly with Bcl2 (Figure 6B). We then tested various cell death-inducing agents that act by stimulating various stress pathways. No compounds were protective against the broad kinase inhibitor, Staurosporine (Figure 6C); the DNA double-strand break-inducing agent, Etoposide (Figure 6D); the Ca-permeablizer, Ionomycin (Figure 6E); or a ER stress inducer, Tunicamycin (Figure 6G). Remarkably however, two-thirds of our hits showed significant protection from tBHP (tert-butyl hydrogen peroxide), an oxidative stress-inducing agent (Figure 6F). Some of these compounds are predicted to be antioxidants based on chemistry (compounds 47 and 52), or similarity to known antioxidants (ethoxyquin, series 2), while the majority lack obvious reducing activity. To further evaluate the anti-oxidative stress activity of these compounds, we tested them against an independent cell type (3 T3 cells) exposed to tBHP (Figure 6H). Compounds that protected from tBHP tended to do so equally well in both cell types (Figure 6I). To better characterize the tBHP protective activity of these compounds, we tested each compound over a range of doses and ordered their activity (Additional file 2: Table S2).

### Activity of the panel of compounds in DUX4-expressing human cells

Finally, to determine whether any of these compounds showed activity in human cells expressing DUX4, we tested the panel of compounds on DUX4-expressing 293T cells. Of the 52 compounds, 32 compounds showed a significant improvement in viability of 293T cells expressing DUX4 (Figure 6J).

## Discussion

FSHD is one of the most common myopathies, with an estimated incidence of 1/20,000 at birth [19,20]. The disease is usually noticed in the teen years, is typically slowly progressive, and because heart and diaphragm are spared, affected individuals live relatively normal lifespans. This means that the prevalence of FSHD is on the same order as the more common but lethal Duchenne muscular dystrophy, and that FSHD patients face a lifetime of dealing with the devastating effects of this muscular dystrophy. There is currently no treatment for FSHD, thus there is an acute and significant need for new medicines in this area. Research on pharmacological inhibitors in FSHD has lagged badly behind advances that have been made over the past decade for many less prevalent genetic diseases. This can be attributed to the genetic complexity of FSHD: the fact that it is not a simple loss or gain of function within a classical gene has led to a variety of poorly-supported, conflicting models, and with the lack of an agreed upon molecular target, a lack of interest in drug development. Although the mechanism by which muscle degeneration occurs is still not well understood, the recent pinpointing of stabilized mRNA encoding DUX4 as essential for FSHD [11], now provides a molecular target in this disease.

Because the effects of DUX4 on C2C12 myoblasts are rapid and easily quantified (cells die in the presence of DUX4), this system enables a highly sensitive cell-based assay. The iC2C12-DUX4 cell line that we have used for these studies is clonal, and the level of DUX4 can be titrated by varying the dose of the inducer, doxycycline. These features provide the system with excellent reproducibility, and therefore allowed us to identify and validate a large set of compounds that protect myoblast cells from the cytotoxic effects of DUX4. The main false positives that we expected were inhibitors of the Tet-on system, and these were effectively identified by using a related cell line, in which luciferase was introduced into the identical genomic locus, regulated by dox in the same manner as DUX4. Although we identified a subset of Tet-on/luciferase inhibitors within our primary hit set, this was not the largest class of false positives. Rather, an unexpected class of compounds inhibiting cell proliferation represented the largest subset. Using a simple serum-titration experiment to test DUX4 toxicity in cells proliferating at different rates, we discovered that the cell-based screen we used gave the greatest differential readout when cells were proliferating most rapidly. We assume that this is due in part to the compounding effect of cell proliferation during the 24 h of exposure to DUX4 in which the screen was performed, and in part to a greater sensitivity of rapidly dividing cells. The final secondary screen that we subjected our hit set to was to test compounds to see whether they also protect 3T3 cells from DUX4. Although FSHD is a disease of muscle, it is not proven that non-myogenic cells have no role, and useful inhibitors ought to protect from the effect of DUX4 on as many cell types as possible. However, in view of the possibility that there may be muscle-specific pathways that might be able to inhibit DUX4 activity, we carefully evaluated the set of compounds eliminated by this final screen, and found that 53 of them were within the identified chemical series, more or less randomly distributed. The lack of C2C12-specific chemical series argues against the idea that these compounds act in muscle-specific inhibitory pathways.

This rigorous secondary screening tree reduced our 1,280 compound hit set to 183 compounds, representing an effective hit rate of 1/240 compounds over the entire set of 44,000 compounds screened. Within the initial 1,280 hit set, we identified 39 chemical series. In some series, the majority of members passed all secondary screens to make it into the 183 compound hit set, while in others, none or only a small subset passed. At this point, assignments to chemical series are highly provisional, and it is likely that many of these series, especially the ones with 20+ members, are grouping compounds based on a feature that is not responsible for activity.

From these 183 compounds, 54 compounds were purchased and retested, and all but two showed activity when tested again in the primary screen. Each of these 52 compounds then, blocks the toxic activity of DUX4 in C2C12 myoblasts. The mechanism of activity of these compounds is not currently known, and because the cell death phenotype against which we screened is likely the product of a multistep pathway, it is probable that these compounds can be further segregated into subclasses based on targeting DUX4 directly, targeting an upstream pathway necessary for DUX4 activity, or targeting different downstream pathways responsible for carrying out DUX4-mediated cell death. To help stratify the compounds we identified into different subgroups, and to gain insight into which pathways were relevant to DUX4-mediated cell death, we tested each compound for the ability to provide protection against six different types of cell death-inducing insults. Remarkably, this identified a large subset of compounds that acts downstream of DUX4 in the oxidative stress pathway. In addition, although they protected only modestly compared to the 52 selected compounds, several of the Prestwick compounds are also annotated as anti-oxidants. Most of the compounds that we selected lack obvious reducing activity, suggesting that they protect from oxidative stress indirectly, probably by stimulating pathways that promote the cells’ own antioxidant defenses. The mechanism of action of each compound bears further study, however given that so many of the protective compounds also protect against oxidative stress, it seems likely that oxidative stress is a major downstream effector pathway of DUX4. Because our library was not directed against a specific pathway, but was indeed random, the fact that the majority of protective compounds also protect against oxidative stress and that no compounds were simple non-specific inhibitors of apoptosis or other cell death insults, it seems that oxidative stress is the critical pathway by which DUX4 is toxic in this system. This is interesting because markers of oxidative stress have been observed in FSHD muscle [21]. In addition, expression profiling in FSHD myoblasts [22] and in cells misexpressing DUX4 [13] suggests that genes involved in oxidative stress response are misregulated. In addition, myoblasts from FSHD patients have been described to be more sensitive to oxidative stress than control myoblasts [23]. Together, these data suggest that protecting cells from oxidative stress, perhaps through one of the inhibitors identified in this study, might be therapeutically beneficial to FSHD patients.

For approximately one-third of the inhibitors we discovered we were not able to identify a pathway downstream of DUX4 in which the inhibitor acted. This could mean that these compounds act on DUX4 itself, or on a pathway required for its activity, for example activity of a kinase that phosphorylates DUX4, or against an as-yet unidentified downstream pathway. Further work will be necessary to identify how these compounds are protective against DUX4, and whether they might be suitable for drug development.

## Conclusion

These studies identify inhibitors of DUX4-mediated toxicity, and demonstrate that such compounds can be used as probes of DUX4 pathology. They support a model in which oxidative stress is an important downstream pathway in the pathology of FSHD.

## Competing interests

The authors declare that they have no competing interests.

## Authors’ contributions

DB developed and carried out the primary and secondary screens and contributed to drafting the manuscript. SHC carried out the analysis of the repurchased compounds and cell death screen and contributed to drafting the manuscript. JMS performed cheminformatics. EAT participated in analysis of the repurchased compounds. MAW performed cheminformatics and contributed to drafting the manuscript. MK conceived of the study, contributed to study design and wrote the manuscript. All authors read and approved the final manuscript.

## Supplementary Material

Additional file 1: Table S1 Dose response analyses for the 52 and Prestwick compounds against DUX4-induced toxicity in iC2C12-DUX4 cells. Click here for file

Additional file 2: Table S2 Activity of the 52 compounds and 4 Prestwick compounds against tBHP-induced toxicity in C2C12 cells. Click here for file
