# Supplementary material for: High-throughput screening identifies inhibitors of DUX4-induced myoblast toxicity
Source: Skelet Muscle. 2014 Feb 1;4:4. doi: 10.1186/2044-5040-4-4 (PMC3914678; doi:10.1186/2044-5040-4-4)
Supplement: Additional file 1: Table S1 — Dose response analyses for the 52 and Prestwick compounds against DUX4-induced toxicity in iC2C12-DUX4 cells. [file 2044-5040-4-4-S1.pdf]

Table S1 Dose response analyses for the 52 and Prestwick compounds against DUX4 induced toxicity in iC2C12-DUX4 cells

Compound ID	Structure	core ID	Concentration							Protection against tBHP	Protection against DUX4 in 293T
			7.44 μM	2.47 μM	0.81 μM	0.27 μM	0.09 μM	0.03 μM	0.01 μM		
1	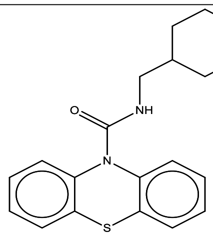 O=C(N1c(c2Sc(c13)cccc3)cccc2)NCC4CCCCC4	4	28.58991	16.07374	31.20957	12.28978	8.796895	0.6468305	0.3557568	Yes	No
2	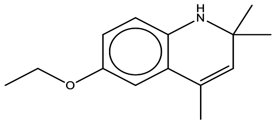 C(C)(=CC1(C)C)c2c(N1)ccc(OCC)c2	2	25.09702	27.71669	29.46313	20.14877	11.12549	2.102199	0.6468305	Yes	Yes
3	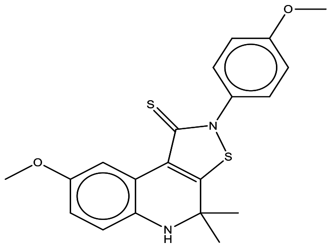 COc1ccc(N2C(=S)C3=C(C(C)(C)Nc(c34)ccc(OC)c4)S2)cc1	2	21.31307	28.00776	20.14877	23.05951	14.03622	2.684347	-0.808538	Yes	No
4	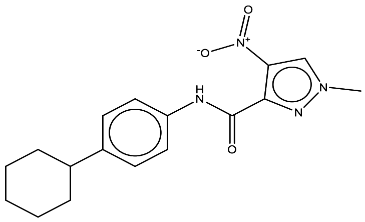 c1(cn(nc1C(=O)Nc(cc2)ccc2C3CCCCC3)C)[N+](=O)[O-]	Singleton	23.78719	27.71669	22.76843	16.65589	5.012937	2.53881	-2.554981	Yes	No
5	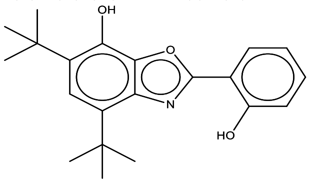 Oc1c(c2nc(c3o2)c(C(C)(C)C)cc(C(C)(C)C)c3O)cccc1	Singleton	13.45407	23.93273	26.26132	25.67917	8.942432	0.3557568	3.266494	Yes	Yes

6	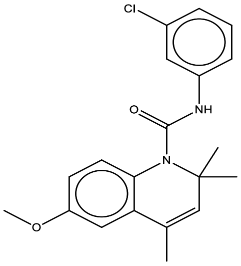 COc1cc2c(N(C(Nc3cc(Cl)ccc3)=O)C(C)(C)C=C2C)cc1	2	25.67917	20.73092	23.93273	22.47736	13.163	5.30401	-0.808538	Yes	Yes
7	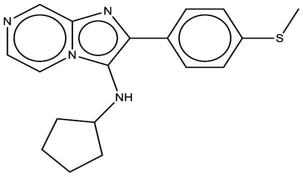 CSc1ccc(c2c(NC3CCCC3)n(c4n2)ccnc4)cc1	12	18.40233	25.09702	14.3273	3.848642	-0.517465	-2.11837	-0.226391	Yes	No
8	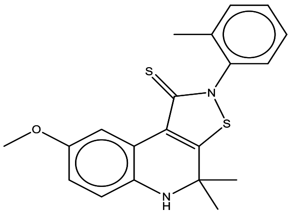 COc1cc(c2cc1)C(=C3C(C)(C)N2)C(=S)N(c4c(C)cccc4)S3	2	24.80595	15.49159	13.45407	19.27555	7.6326	5.30401	1.811126	Yes	No
9	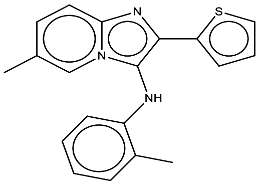 Cc1cn(c2cc1)c(Nc3c(C)cccc3)c(c4sccc4)n2	1	24.2238	13.163	6.177232	-3.137128	-1.827296	-3.719275	-0.808538	Yes	Yes
10	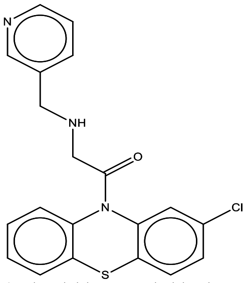 Clc1cc(c2cc1)N(C(CNCc3cnccc3)=O)c(c4S2)cccc4	4	21.16753	24.2238	17.38357	11.99871	5.740621	0.3557568	3.848642	Yes	No

11	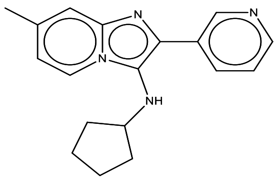 Cc1cc2n(c(NC3CCCC3)c(c4cnccc4)n2)cc1	1	23.64165	9.087969	5.158473	2.684347	-4.592497	-1.390686	-2.554981	No	Yes
12	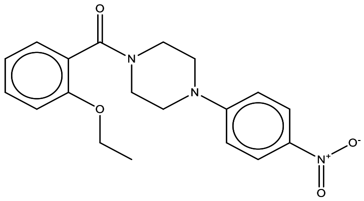 CCOc1c(C(N2CCN(c3ccc([N+]([O-])=O)cc3)CC2)=O)cccc1	Singleton	22.47736	23.64165	21.31307	16.80142	5.740621	1.520052	-2.846054	No	No
13	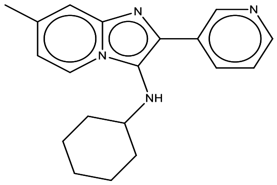 Cc1cc2n(c(NC3CCCCC3)c(c4cnccc4)n2)cc1	1	21.31307	23.64165	20.14877	8.505821	-0.808538	-2.409444	0.3557568	No	No
14	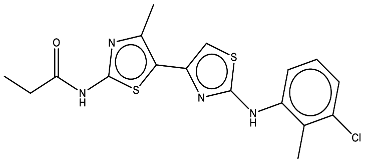 CCC(Nc1sc(c2nc(Nc3c(C)c(Cl)ccc3)sc2)c(C)n1)=O	13	20.87646	22.47736	17.82018	10.10673	0.3557568	-1.536222	0.9379042	Yes	No
15	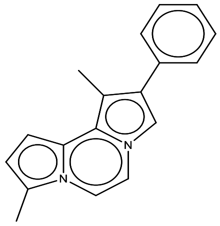 Cc1c(n2cc1c3ccccc3)c(n4cc2)ccc4C	Singleton	21.02199	22.47736	15.78267	13.74515	0.3557568	1.520052	-0.808538	Yes	No

16	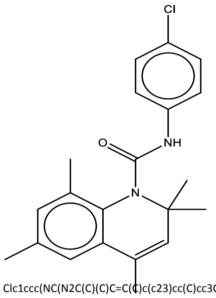 Clc1ccc(NC(N2C(C)(C)C=C(C)c(c23)cc(C)cc3C)=O)cc1	2	20.43984	20.14877	22.04075	16.65589	8.796895	0.7923674	0.7923674	Yes	No
17	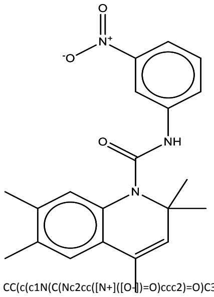 CC(c(c1N(C(Nc2cc([N+]([O-])=O)ccc2)=O)C3(C)C)cc(C)c(C)c1)=C3	2	18.54786	17.23803	21.89521	16.07374	15.49159	5.30401	6.177232	Yes	No
18	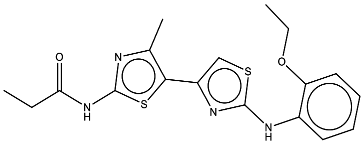 CCOc1c(Nc2nc(c3c(C)nc(NC(CC)=O)s3)cs2)cccc1	13	21.60414	17.23803	5.30401	1.083441	-0.226391	1.520052	-0.517465	No	Yes
19	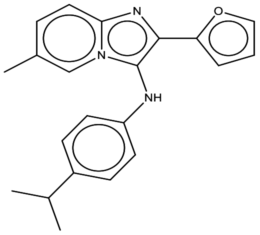 Cc1cn(c2cc1)c(Nc3ccc(C(C)C)cc3)c(c4occc4)n2	1	12.58085	12.28978	15.49159	15.49159	21.31307	18.40233	21.02199	No	Yes
20	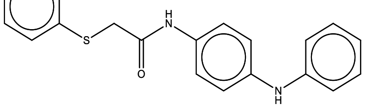 O=C(Nc1ccc(Nc2ccccc2)cc1)CSc3ccccc3	Singleton	20.43984	21.31307	12.14424	3.412031	-2.846054	-0.808538	-1.827296	Yes	Yes

21	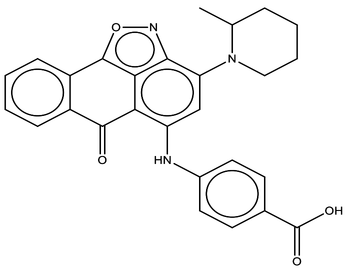<br>OC(c1ccc(Nc2c(c(c3on4)c4c(N5C(C)CCCC5)c2)C(=O)c(c36)cccc6)cc1)=O	26	21.31307	18.11126	9.52458	1.520052	0.0646831	-3.573739	0.0646831	No	Yes
22	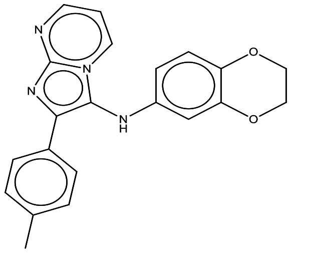<br>n1(cccn2)c2nc(c3ccc(cc3)C)c1Nc(ccc(c45)OCCO4)c5	3	21.02199	14.3273	2.102199	-1.390686	-1.245149	-5.320181	-0.517465	No	Yes
23	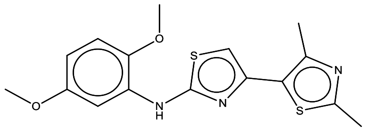<br>COc1c(Nc2nc(c3c(C)nc(C)s3)cs2)cc(OC)cc1	14	15.78267	21.02199	5.595084	-1.390686	-6.047865	-5.465718	-2.700517	Yes	No
24	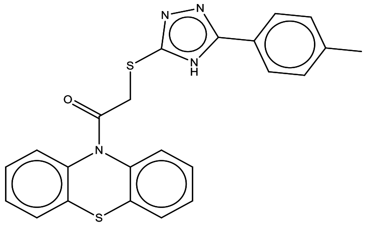<br>N(C(=O)CSc1nnc(c2ccc(cc2)C)[nH]1)(c3c4cccc3)c5c(S4)cccc5	6	18.40233	20.73092	15.9282	11.27102	4.139715	-1.681759	0.6468305	Yes	Yes
25	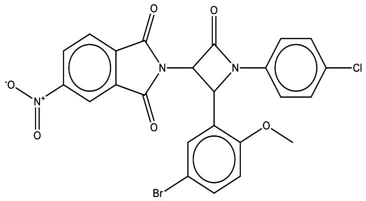<br>COc1c(C2C(N3C(=O)c(c4C3=O)cc([N+]([O-])=O)cc4)C(=O)N2c5ccc(Cl)cc5)c	Singleton	18.40233	15.49159	20.73092	20.58538	11.85317	8.505821	15.49159	Yes	Yes

26	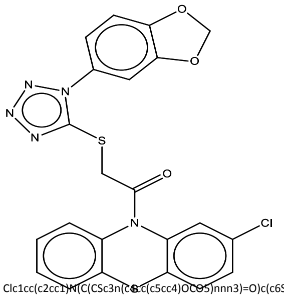 Clc1cc(c2cc1)N(C(CSc3n(c4cc(c5cc4)OCO5)nnn3)=O)c(c6S2)cccc6	6	15.49159	20.58538	10.83441	-0.371928	-0.808538	-4.44696	-0.808538	No	Yes
27	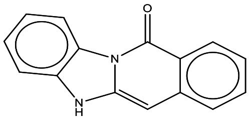 O=C1N(C2=Cc(c13)cccc3)c(c4N2)cccc4	Singleton	20.58538	15.49159	10.83441	1.811126	0.2102199	0.3557568	-3.137128	Yes	Yes
28	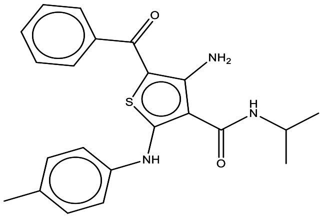 Cc1ccc(Nc2c(C(NC(C)C)=O)c(N)c(C(c3ccccc3)=O)s2)cc1	Singleton	14.61837	20.43984	14.90944	-2.263907	-3.137128	-3.137128	-2.409444	No	Yes
29	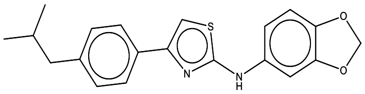 n1c(c2ccc(CC(C)C)cc2)csc1Nc3ccc(c4c3)OCO4	7	15.49159	20.43984	19.56662	18.11126	13.59961	6.613842	3.412031	Yes	Yes
30	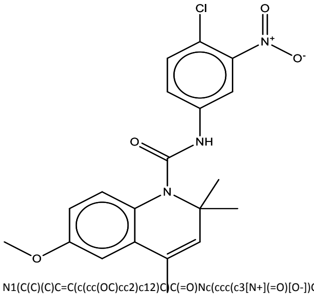 N1(C(C)(C)C=C(c(cc(OC)cc2)c12)C)C(=O)Nc(ccc(c3[N+](=O)[O-])Cl)c3	2	18.98448	19.13001	17.82018	20.43984	8.796895	1.520052	0.9379042	Yes	Yes

31	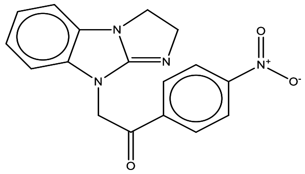 C1(=NCC2)N2c(c3N1CC(=O)c(cc4)ccc4[N+](=O)[O-])cccc3	Singleton	20.14877	13.74515	5.30401	-1.390686	0.9379042	-4.44696	-0.226391	No	No
32	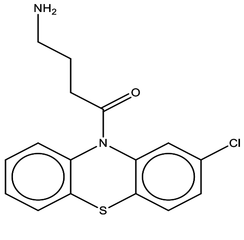 NCCCC(N1c(c2Sc(c13)cccc3)cc(Cl)cc2)=O	4	11.5621	14.47283	20.14877	20.14877	11.99871	2.393273	1.956662	No	No
33	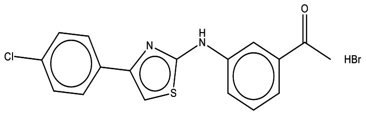 Br.Clc1ccc(c2nc(Nc3cc(C(C)=O)ccc3)sc2)cc1	7	19.71216	17.82018	7.6326	1.811126	-2.554981	-2.11837	-5.029108	No	No
34	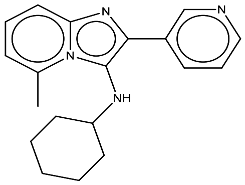 Cc1n(c2ccc1)c(NC3CCCCC3)c(c4cnccc4)n2	1	14.3273	14.47283	13.163	7.050453	13.45407	19.56662		No	Yes
35	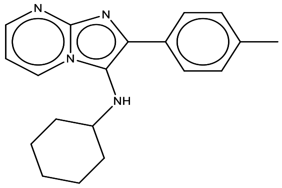 Cc1ccc(c2c(NC3CCCCC3)n(c4n2)cccn4)cc1	3	18.98448	13.59961	7.341527	0.0646831	-2.554981	-4.592497	-2.700517	No	No

36	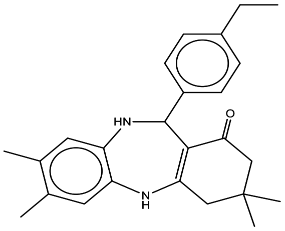 CCc1ccc(C2C3=C(CC(C)(C)CC3=O)Nc(c4N2)cc(C)c(C)c4)cc1	8	17.82018	14.61837	0.6468305	-4.155886	-5.029108	-0.808538	-0.808538	Yes	Yes
37	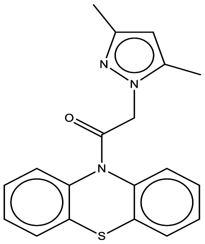 Cc1cc(C)n(CC(N2c(c3Sc(c24)cccc4)cccc3)=O)n1	4	17.82018	16.07374	5.449547	-5.029108	-3.573739	-2.554981	-2.846054	Yes	Yes
38	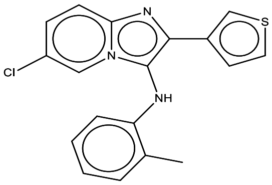 Clc1cn(c2cc1)c(Nc3c(C)cccc3)c(c4cscc4)n2	1	17.0925	7.923674	-0.226391	-4.155886	-1.972833	6.177232		Yes	Yes
39	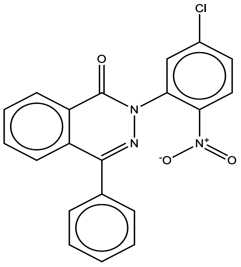 Clc1cc(N2C(=O)c(c3C(c4ccccc4)=N2)cccc3)c([N+]([O-])=O)cc1	Singleton	16.36481	10.3978	-0.517465	0.2102199	4.8674	14.03622		Yes	Yes
40	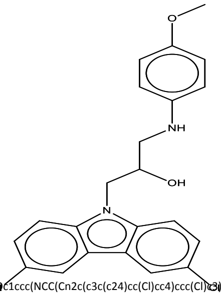 COc1ccc(NCC(Cn2c(c3c(c24)cc(Cl)cc4)ccc(Cl)c3)O)cc1	Singleton	14.61837	16.36481	14.03622	13.163	1.520052	-1.681759	-0.371928	Yes	Yes

41	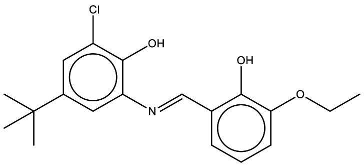 CCOc1c(O)c(\C=N\c2c(O)c(Cl)cc(C(C)(C)C)c2)ccc1	11	16.36481	12.43532	5.30401	-1.972833	-0.226391	-2.409444	-0.808538	Yes	Yes
42	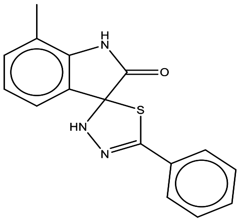 Cc1c2c(C3(SC(c4ccccc4)=NN3)C(=O)N2)ccc1	Singleton	16.07374	16.07374	4.139715	-0.226391	-0.663001	-0.808538	-2.846054	No	No
43	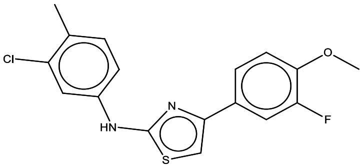 n1c(c2ccc(OC)c(F)c2)csc1Nc3ccc(C)c(Cl)c3	7	12.72639	13.45407	15.49159	5.158473	4.139715	8.796895		Yes	No
44	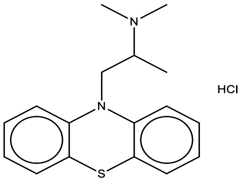 Cl.CN(C(CN1c(c2Sc(c13)cccc3)cccc2)C)C	4	5.012937	13.45407	15.49159	3.266494	-1.972833	-1.681759	-0.808538	Yes	No
45	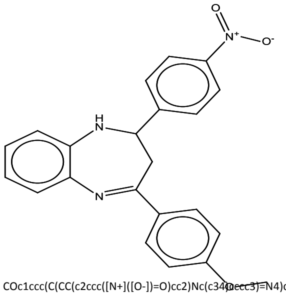 COc1ccc(C(CC(c2ccc([N+]([O-])=O)cc2)Nc(c34)cccc3)=N4)cc1	Singleton	15.27849	9.505571	2.33473	2.082327	2.23118	0.2443134	-0.292853	Yes	Yes

46	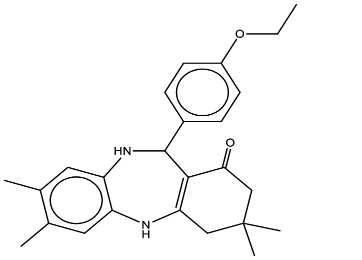 CCOc1ccc(C2C3=C(CC(C)(C)CC3=O)Nc(c4N2)cc(C)c(C)c4)cc1	8	11.70763	10.97995	6.177232	6.468305	10.97995	14.90944		Yes	Yes
47	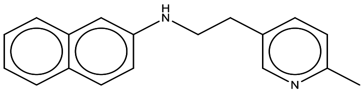 Cc1ncc(CCNc2cc(c3cc2)cccc3)cc1	Singleton	10.97995	14.3273	13.163	0.5012937	-2.554981	-4.010349	-3.137128	No	Yes
48	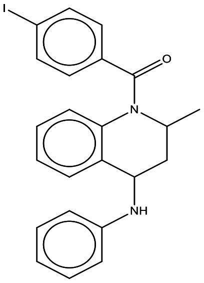 Ic1ccc(C(N2c(c3C(Nc4ccccc4)CC2C)cccc3)=O)cc1	Singleton	14.3273	9.96119	0.9379042	1.520052	-1.827296	-1.681759	-0.663001	Yes	Yes
49	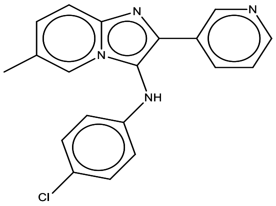 Clc1ccc(Nc2n(c3nc2c4cnccc4)cc(C)cc3)cc1	1	13.74515	1.811126	0.7923674	-5.029108	-5.465718	-3.573739	-0.808538	No	Yes
α-Tocopherol			11.99871	6.759379	-0.226391	-0.808538	-2.700517	-4.44696	-0.226391	No	

50	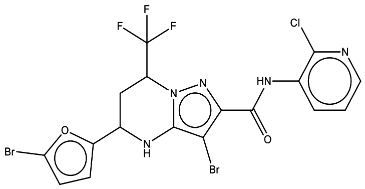 Brc1oc(C2CC(C(F)(F)F)n(c3N2)nc(C(Nc4c(Cl)nccc4)=O)c3Br)cc1	Singleton	10.59932	6.301992	-2.312079	-1.930238	-2.189113	-1.49015	0.9885795	No	Yes
51	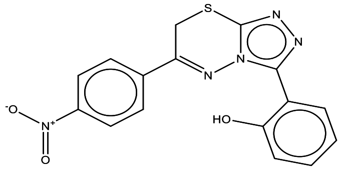 Oc1c(c2n(c3nn2)N=C(c4ccc([N+]([O-])=O)cc4)CS3)cccc1	Singleton	8.505821	1.083441	-3.573739	-4.010349	-5.320181	-3.137128	-4.010349	No	No
Vitamin K2			3.120957	-2.263907	-0.517465	-0.371928	-0.371928	0.0646831			
Ethoxyquin			1.739317	3.09194	-1.412488	0.4967167	-0.331684	-1.496622	-0.370515		
Ethopropazine hydrochloride			2.451224	1.389836	-0.771772	-0.435234	-1.393072	0.2184259	-0.325212		

52	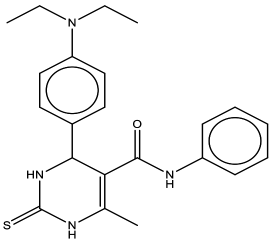 CCN(c1ccc(C2C(C(Nc3ccccc3)=O)=C(C)NC(=S)N2)cc1)CC	Singleton	-1.245149	-0.517465	-0.517465	1.811126	-1.681759	2.102199	-1.390686	No	No
