# Supplementary material for: High-throughput screening identifies inhibitors of DUX4-induced myoblast toxicity
Source: Skelet Muscle. 2014 Feb 1;4:4. doi: 10.1186/2044-5040-4-4 (PMC3914678; doi:10.1186/2044-5040-4-4)
Supplement: Additional file 2: Table S2 — Activity of the 52 compounds and 4 Prestwick compounds against tBHP-induced toxicity in C2C12 cells. [file 2044-5040-4-4-S2.pdf]

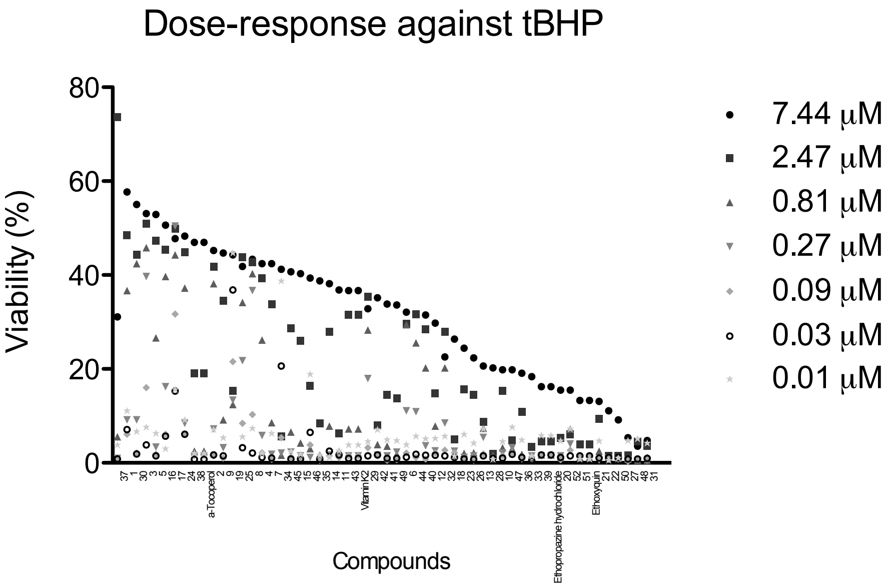

Table S2. Dose response analyses for the 52 and Prestwick compounds against tBHP induced toxicity in C2C12 cells

Compound ID	core ID	Concentration						
		7.44 µM	2.47 µM	0.81 µM	0.27 µM	0.09 µM	0.03 µM	0.01 µM
37	4	31.13233	73.72442	5.538881	0.9549795	0.7639836	0.8594816	3.819918
1	4	57.68076	48.51296	36.67122	9.167804	6.111869	7.066849	11.07776
30	2	55.00682	44.31105	42.40109	9.167804	2.100955	1.909959	6.684857
3	2	53.09686	50.99591	45.83902	39.72715	16.04366	3.819918	7.639836
5	Singleton	52.90586	47.36699	26.64393	3.342428	1.814461	1.527967	6.302865
16	2	50.61391	45.45703	39.72715	16.23465	6.111869	5.729877	3.055934
17	2	47.74898	49.84993	44.31105	50.42292	31.70532	15.27967	15.66166
24	6	48.32196	44.88404	37.2442	8.40382	6.111869	6.111869	9.167804
38	1	46.98499	19.09959	2.482947	1.527967	1.623465	0.7639836	2.005457
α-Tocopherol		46.98499	19.09959	2.482947	1.527967	1.623465	0.7639836	2.005457
2	2	45.26603	41.8281	38.19918	7.257844	1.527967	1.718963	7.066849
9	1	44.69304	34.57026	9.167804	3.24693	1.050478	1.527967	5.347886
19	1	44.31105	15.27967	12.41473	13.36971	21.58254	36.86221	44.69304
25	Singleton	41.8281	43.73806	34.18827	21.77353	8.40382	3.24693	5.538881
8	2	43.35607	42.78308	40.30014	36.67122	10.31378	2.100955	7.353343
4	Singleton	42.40109	39.34516	26.16644	5.825375	2.196453	1.145975	2.100955
7	12	42.40109	33.80627	8.594816	1.814461	0.7639836	1.050478	6.302865
34	1	41.25512	5.729877	1.623465	2.100955	5.347886	20.62756	38.77217
45	Singleton	40.68213	28.64939	6.589359	2.291951	1.241473	0.8594816	2.482947
15	Singleton	40.30014	25.97544	4.20191	1.527967	1.145975	0.8594816	1.814461
46	8	39.34516	16.42565	3.055934	1.145975	3.819918	6.493861	18.90859
35	3	38.77217	8.40382	1.718963	0.5729877	0.5729877	0.7639836	1.623465
14	13	38.19918	27.8854	7.830832	1.718963	1.527967	2.482947	1.241473
11	1	36.86221	6.302865	2.482947	1.050478	1.145975	1.718963	2.578445
43	7	36.67122	31.51432	7.257844	1.527967	1.527967	0.9549795	3.819918
Vitamin K2		36.67122	31.51432	7.257844	1.527967	1.527967	0.9549795	3.819918
29	7	32.8513	35.33424	28.2674	17.95362	3.24693	1.527967	4.583902
42	Singleton	35.14325	8.021828	3.915416	1.623465	1.527967	1.718963	7.066849
41	11	33.80627	14.51569	3.533424	1.527967	0.5729877	0.9549795	4.965894
49	1	33.61528	13.75171	1.814461	2.291951	0.6684857	0.9549795	4.774898
6	2	32.08731	29.60437	29.41337	11.07776	2.100955	1.241473	3.915416
44	4	31.70532	31.70532	25.59345	10.88677	1.145975	1.814461	5.729877
40	Singleton	31.51432	28.45839	20.24557	3.628922	0.8594816	1.623465	5.156889
12	Singleton	29.79536	14.80218	7.830832	2.578445	2.100955	1.623465	5.252388

32	4	22.53752	27.8854	20.24557	8.499318	2.673943	1.527967	5.729877
18	13	26.35744	5.061391	1.718963	2.482947	2.196453	1.145975	1.527967
23	14	24.44748	15.66166	2.005457	1.145975	0.9549795	0.8594816	6.111869
26	6	22.34652	14.51569	2.196453	1.527967	0.5729877	0.7639836	4.20191
13	1	20.62756	8.785811	7.353343	5.443383	1.909959	1.527967	7.257844
28	Singleton	20.24557	1.909959	1.718963	0.7639836	0.8594816	0.9549795	0.7639836
10	4	19.86357	15.37517	3.24693	2.100955	1.527967	1.050478	4.583902
47	Singleton	19.86357	4.774898	3.055934	3.24693	2.196453	1.814461	7.639836
36	8	19.09959	10.88677	1.814461	0.7639836	1.241473	1.145975	4.870396
33	7	18.33561	3.437926	0.9549795	1.527967	1.241473	0.4774898	1.527967
39	Singleton	16.23465	4.583902	1.718963	1.527967	1.718963	1.718963	5.825375
Ethopropazine hydrochloride		16.23465	4.583902	1.718963	1.527967	1.718963	1.718963	5.825375
20	Singleton	15.47067	5.347886	1.145975	1.527967	0.6684857	1.145975	4.679399
52	Singleton	15.47067	6.111869	7.257844	4.010914	2.673943	1.527967	7.257844
51	Singleton	13.27422	3.915416	1.145975	0.9549795	1.241473	1.527967	0.8594816
Ethoxyquin		13.27422	3.915416	1.145975	0.9549795	1.241473	1.527967	0.8594816
21	26	13.08322	9.358799	2.482947	0.5729877	0.8594816	1.050478	4.679399
22	3	11.07776	1.527967	1.241473	1.050478	0.7639836	1.145975	0.5729877
50	Singleton	9.167804	1.527967	0.9549795	0.7639836	1.050478	1.145975	1.050478
27	Singleton	5.347886	1.623465	0.7639836	0.6684857	0.3819918	0.8594816	4.774898
48	Singleton	3.533424	4.583902	0.5729877	0.4774898	0.7639836	0.8594816	5.156889
31	Singleton	4.774898	3.628922	0.5729877	0.4774898	1.145975	0.9549795	4.20191
